# Supplementary material for: In Vitro Activity of “Old” and “New” Antimicrobials against the Klebsiella pneumoniae Complex
Source: Antibiotics (Basel). 2024 Jan 26;13(2):126. doi: 10.3390/antibiotics13020126 (PMC10886291; doi:10.3390/antibiotics13020126)
Supplement: Supplementary file 1 [file antibiotics-13-00126-s001.zip › antibiotics-2807632-supplementary.pdf]

Table S1: Data refer to susceptibility of *K. pneumoniae complex* (*n* = 160) obtained from Phoenix (according to EUCAST 2023)

**No.**	**Species**	**Susceptibility**
1	*K. pneumoniae*	AN
2	*K. pneumoniae*	SXT
3	*K. pneumoniae*	-
4	*K. pneumoniae*	-
5	*K. pneumoniae*	-
6	*K. pneumoniae*	-
7	*K. pneumoniae*	-
8	*K. pneumoniae*	-
9	*K. pneumoniae*	-
10	*K. pneumoniae*	MEM, AN
11	*K. pneumoniae*	GM, NN, AN
12	*K. pneumoniae*	GM, SXT
13	*K. pneumoniae*	GM, AN, SXT
14	*K. pneumoniae*	GM, AN
15	*K. pneumoniae*	AN
16	*K. pneumoniae*	AN
17	*K. pneumoniae*	AN
18	*K. pneumoniae*	AN
19	*K. pneumoniae*	AN
20	*K. pneumoniae*	AN
21	*K. pneumoniae*	AN
22	*K. pneumoniae*	AN
23	*K. pneumoniae*	AN
24	*K. pneumoniae*	AN
25	*K. pneumoniae*	AN
26	*K. pneumoniae*	AN
27	*K. pneumoniae*	GM, NN, AN
28	*K. pneumoniae*	GM, AN, NN
29	*K. pneumoniae*	GM, SXT
30	*K. pneumoniae*	GM, SXT
31	*K. pneumoniae*	GM, SXT
32	*K. pneumoniae*	GM
33	*K. pneumoniae*	GM, SXT
34	*K. pneumoniae*	GM, SXT
35	*K. pneumoniae*	GM
36	*K. pneumoniae*	GM
37	*K. pneumoniae*	GM, SXT
38	*K. pneumoniae*	GM, SXT
39	*K. pneumoniae*	GM
40	*K. pneumoniae*	GM, SXT
41	*K. pneumoniae*	GM, AN, SXT
42	*K. pneumoniae*	AN
43	*K. pneumoniae*	-
44	*K. pneumoniae*	GM, SXT
45	*K. pneumoniae*	GM
46	*K. pneumoniae*	-
47	*K. pneumoniae*	GM, AN
48	*K. pneumoniae*	GM, AN, SXT
49	*K. pneumoniae*	SXT
50	*K. pneumoniae*	-
51	*K. pneumoniae*	-
52	*K. pneumoniae*	-
53	*K. pneumoniae*	-

54	*K. pneumoniae*	GM, AN, SXT
55	*K. pneumoniae*	-
56	*K. pneumoniae*	AN
57	*K. pneumoniae*	-
58	*K. pneumoniae*	-
59	*K. pneumoniae*	-
60	*K. pneumoniae*	-
61	*K. pneumoniae*	-
62	*K. pneumoniae*	-
63	*K. pneumoniae*	GM, AN, NN
64	*K. pneumoniae*	GM, AN
65	*K. pneumoniae*	-
66	*K. pneumoniae*	-
67	*K. pneumoniae*	GM
68	*K. pneumoniae*	AN
69	*K. pneumoniae*	GM, AN
70	*K. pneumoniae*	AN
71	*K. pneumoniae*	AN
72	*K. pneumoniae*	GM, AN
73	*K. pneumoniae*	AN
74	*K. pneumoniae*	AN
75	*K. pneumoniae*	GM
76	*K. pneumoniae*	AN, CIP, LEV, SXT
77	*K. pneumoniae*	-
78	*K. pneumoniae*	-
79	*K. pneumoniae*	GM, AN
80	*K. pneumoniae*	-
81	*K. pneumoniae*	GM, AN
82	*K. pneumoniae*	-
83	*K. pneumoniae*	AN
84	*K. pneumoniae*	-
85	*K. pneumoniae*	-
86	*K. pneumoniae*	-
87	*K. pneumoniae*	-
88	*K. pneumoniae*	-
89	*K. pneumoniae*	-
90	*K. pneumoniae*	-
91	*K. pneumoniae*	-
92	*K. pneumoniae*	-
93	*K. pneumoniae*	-
94	*K. pneumoniae*	-
95	*K. pneumoniae*	-
96	*K. pneumoniae*	-
97	*K. pneumoniae*	GM
98	*K. pneumoniae*	IPM, MEM, AN
99	*K. pneumoniae*	IPM, AN, SXT
100	*K. pneumoniae*	IPM, MEM, AN
101	*K. pneumoniae*	IPM, MEM, GM
102	*K. pneumoniae*	NN, AN
103	*K. pneumoniae*	IPM, MEM, GM
104	*K. pneumoniae*	IPM, AN
105	*K. pneumoniae*	IPM, MEM, GM
106	*K. pneumoniae*	IPM, MEM, AN
107	*K. pneumoniae*	IPM, MEM, AN
108	*K. pneumoniae*	IPM, MEM, GM
109	*K. pneumoniae*	IPM, AN, SXT
110	*K. pneumoniae*	IPM, MEM, GM

111	*K. pneumoniae*	IPM, MEM, AN
112	*K. pneumoniae*	IPM, MEM, GM
113	*K. pneumoniae*	IPM, MEM, GM, AN
114	*K. pneumoniae*	IPM, MEM, GM, AN, NN
115	*K. pneumoniae*	IPM, MEM, GM, CIP, LEV
116	*K. pneumoniae*	IPM, MEM, GM, AN, NN
117	*K. pneumoniae*	IPM, MEM, GM
118	*K. pneumoniae*	IPM, MEM, GM, AN, NN, SXT
119	*K. pneumoniae*	IPM, MEM, GM
120	*K. pneumoniae*	IPM, MEM, GM, AN
121	*K. pneumoniae*	IPM, MEM, GM, AN
122	*K. pneumoniae*	GM, AN
123	*K. pneumoniae*	IPM, MEM, GM
124	*K. pneumoniae*	IPM, MEM, AN
125	*K. pneumoniae*	AN
126	*K. pneumoniae*	IPM
127	*K. pneumoniae*	-
128	*K. pneumoniae*	GM
129	*K. pneumoniae*	IPM, GM, CIP, LEV
130	*K. pneumoniae*	IPM, MEM
131	*K. pneumoniae*	GM, AN
132	*K. pneumoniae*	-
133	*K. pneumoniae*	IPM, GM, AN
134	*K. pneumoniae*	AN
135	*K. pneumoniae*	IPM, AN
136	*K. pneumoniae*	GM, AN, NN, CIP, LEV, SXT
137	*K. pneumoniae*	GM, AN
138	*K. pneumoniae*	IPM, MEM
139	*K. variicola*	IPM, MEM, GM, AN
140	*K. variicola*	TZP, IPM, MEM, GM
141	*K. variicola*	AN
142	*K. variicola*	IPM, MEM, AN, CIP, LEV
143	*K. variicola*	IPM, MEM, AN, CIP, LEV
144	*K. variicola*	IPM, MEM, GM, AN
145	*K. variicola*	AN, CIP, LEV
146	*K. variicola*	IPM, MEM
147	*K. variicola*	IPM, MEM, GM, AN, SXT
148	*K. variicola*	IPM, MEM, GM, AN, NN, SXT
149	*K. variicola*	IPM, MEM, AN
150	*K. variicola*	IPM, MEM, GM, AN
151	*K. variicola*	IPM, MEM, AN
152	*K. variicola*	FEP, IPM, MEM, GM, AN
153	*K. variicola*	CAZ, IPM, MEM, AN, SXT
154	*K. variicola*	IPM, MEM, AN, CIP, LEV
155	*K. variicola*	IPM, MEM, GM, AN, SXT
156	*K. variicola*	IPM, MEM, GM, AN, SXT
157	*K. variicola*	IPM, MEM, GM, AN, SXT
158	*K. variicola*	IPM, MEM, GM, AN, NN
159	*K. variicola*	IPM, MEM, GM, AN
160	*K. variicola*	IPM, MEM, GM, AN

AN – amikacin, CAZ – ceftazidim, CIP – ciprofloxacin, GM – gentamicin , IPM – imipenem, LEV – levofloxacin, MEM – meropenem, NN – tobramycin, SXT – cotrimoxazole, TZP – piperacillin/tazobactam, „-” – not susceptible to any antimicrobials included in NMIC-408 panel
